# Urban-Rural and Demographic Differences in Substance Use Among High School Students — Youth Risk Behavior Survey, United States, 2025

**DOI:** 10.15585/mmwr.su7501a6

**Published:** 2026-10-15

**Authors:** Sanjana Pampati, Izraelle I. McKinnon, Joohyun Park, Catherine N. Rasberry, Macarena C. García, Nicolas A. Suarez, Emiko Petrosky, Loren F. Thomas, Marci Hertz

**Affiliations:** ^1^Division of Adolescent and School Health, National Center for Chronic Disease Prevention and Health Promotion, CDC; ^2^Division of Overdose Prevention, National Center for Injury Prevention and Control, CDC; ^3^Office of Rural Health, Public Health Infrastructure Center, CDC; ^4^U.S. Public Health Service, Rockville, Maryland

## Abstract

Adolescent substance use remains a critical public health concern. Understanding patterns of substance use might guide prevention strategies and identify populations in need of focused, tailored interventions. This report links nationally representative 2025 Youth Risk Behavior Survey data with school characteristics data to describe substance use behaviors and differences by demographic characteristics and among students in public schools for whom locale information was available, by urban-rural classification. Current (i.e., past 30-day) measures included prescription opioid misuse and use of cigarettes, cigars, smokeless tobacco, electronic vapor products, alcohol, and marijuana. Lifetime measures included prescription opioid misuse and use of electronic vapor products, marijuana, cocaine, inhalants, heroin, methamphetamines, ecstasy, hallucinogenic drugs, a composite of selected illicit drugs, and injection of an illegal drug. Differences by sex, race and ethnicity, grade, and school urban-rural classification were analyzed. Adjusted prevalence ratios were used to assess urban-rural differences after controlling for sex, race and ethnicity, and grade. The most commonly reported substance use behaviors were lifetime use of electronic vapor products (30.2%), followed by marijuana (25.5%) and current use of alcohol (20.3%) and electronic vapor products (16.0%). Substance use varied by sex and race and ethnicity, with differences observed in patterns across behaviors. Most observed grade-related differences reflected higher prevalence among students in higher grade levels. After adjustment, students in rural public schools had a higher prevalence of current smokeless tobacco and electronic vapor product use, as well as lifetime electronic vapor product use, than students in city and suburb or town public schools. In addition, students in rural public schools had a higher prevalence of current cigarette and cigar use than students in suburb or town public schools. These descriptive findings might guide comprehensive and multicomponent youth substance use prevention programs that engage schools, families, and communities, including tobacco and nicotine prevention strategies tailored to rural schools and communities.

## Introduction

Substance use is a critical public health challenge (*1*). Data from the 2024 National Survey on Drug Use and Health found that among persons in the United States aged ≥12 years, 58.3% reported using tobacco products, nicotine vaping products, alcohol, or illicit drugs in the past month (*2*). Although substance use is an issue for both adolescents and adults, research has identified adolescent substance use as a key risk factor for continued substance use in adulthood (*1*). Adolescence is also a period of development during which the brain is particularly sensitive to the effects of drugs, and areas of the brain involved in impulse control, judgment, and decision-making are not yet fully developed (*3*). Youths who engage in substance use during adolescence are at greater risk for substance use disorders, which are associated with increased mental health challenges and engagement in other risk behaviors (*1*,*3*).

Understanding patterns of substance use among adolescents is essential for planning and implementing effective public health responses. Demographic differences in substance use behaviors by sex, race and ethnicity, and grade have been previously identified (*4*) (https://youthonline.cdc.gov). Geographic differences have also been identified, including urban-rural differences in the prevalence of substance use (*5*). Among adolescents, social and environmental factors associated with substance use behaviors, including peer substance use and accessibility of prevention and treatment resources (*1*), might vary across geographic contexts. Data from the national Youth Risk Behavior Survey (YRBS) provide an important opportunity to understand adolescent substance use, including how patterns might differ based on specific substances (e.g., alcohol, tobacco, or other drugs) and to examine how patterns vary by demographic characteristics. YRBS has been used to monitor adolescent substance use for more than 3 decades. More recently, linkage of national YRBS data with school-level characteristics has expanded this surveillance capacity by enabling examination of substance use patterns by urban-rural school classification using nationally representative YRBS data. This report describes patterns of substance use among U.S. high school students and examines differences by sex, race and ethnicity, grade, and urban-rural classification. The findings in this report might guide policymakers, school administrators, and public health officials in the development and implementation of public health and education interventions to address youth substance use.

## Methods

### Data Source

This report includes data from the 2025 YRBS (N = 10,710), a cross-sectional, school-based survey conducted biennially since 1991. Each survey year, CDC collects data from a nationally representative sample of students from public and private schools, including parochial schools, in grades 9–12 in the 50 U.S. states and the District of Columbia. Additional information about YRBS sampling, data collection, response rates, and processing is available in the overview report of this supplement (*6*). The prevalence estimates for all YRBS variables among U.S. high school students overall and stratified by demographic characteristics are available at https://youthonline.cdc.gov. The full YRBS questionnaire, datasets, and documentation are available at https://www.cdc.gov/yrbs/index.html. This study was approved by institutional review boards at CDC and ICF, the survey contractor, and was conducted consistent with applicable federal law and CDC policy.*

YRBS data were linked to 2024–2025 school characteristics data from the National Center for Education Statistics (NCES) Education Demographic and Geographic Estimates Program (https://nces.ed.gov/programs/edge/Geographic/SchoolLocations) to obtain a school-level measure of urban-rural classification. Linkage was based on the NCES school ID, a unique identification number assigned to schools. These data are used and maintained internally by CDC to protect the confidentiality of school-identifying information.

### Measures

Questions, response options, and analytic coding for all substance use behaviors are provided (Table 1). The seven current substance use behaviors, defined as using the substance at least once during the 30 days before the survey, included prescription opioid misuse and use of cigarettes, cigars, smokeless tobacco, electronic vapor products, alcohol, and marijuana. The 11 lifetime substance use behaviors, defined as ever using the substance at least once, included prescription opioid misuse, injection of an illegal drug, and use of electronic vapor products, marijuana, cocaine, inhalants, heroin, methamphetamines, ecstasy, hallucinogenic drugs, and a composite of selected illicit drugs (i.e., cocaine, inhalants, heroin, methamphetamines, ecstasy, and hallucinogenic drugs).

**TABLE 1:** Questions, response options, and analytic coding for current and lifetime use of specific substances among high school students — Youth Risk Behavior Survey, United States, 2025

Variable	Question	Response option	Analytic coding
**Current use**			
Cigarettes	During the past 30 days, on how many days did you smoke cigarettes?	0 days, 1 or 2 days, 3–5 days, 6–9 days, 10–19 days, 20–29 days, or all 30 days	Yes: 1 or 2 days, 3–5 days, 6–9 days, 10–19 days, 20–29 days, or all 30 days No: 0 days
Cigars	During the past 30 days, on how many days did you smoke cigars, cigarillos, or little cigars, such as Swisher Sweets, Middleton’s (including Black and Mild), or Backwoods?	0 days, 1 or 2 days, 3–5 days, 6–9 days, 10–19 days, 20–29 days, or all 30 days	Yes: 1 or 2 days, 3–5 days, 6–9 days, 10–19 days, 20–29 days, or all 30 days No: 0 days
Smokeless tobacco	During the past 30 days, on how many days did you use chewing tobacco, snuff, dip, snus, dissolvable tobacco products, or nicotine pouches such as Copenhagen, Grizzly, Skoal, Camel Snus, on!, ZYN, or Velo? (Do not count any electronic vapor products.)	0 days, 1 or 2 days, 3–5 days, 6–9 days, 10–19 days, 20–29 days, or all 30 days	Yes: 1 or 2 days, 3–5 days, 6–9 days, 10–19 days, 20–29 days, or all 30 days No: 0 days
Electronic vapor products	During the past 30 days, on how many days did you use an electronic vapor product?	0 days, 1 or 2 days, 3–5 days, 6–9 days, 10–19 days, 20–29 days, or all 30 days	Yes: 1 or 2 days, 3–5 days, 6–9 days, 10–19 days, 20–29 days, or all 30 days No: 0 days
Alcohol	During the past 30 days, on how many days did you have at least one drink of alcohol?	0 days, 1 or 2 days, 3–5 days, 6–9 days, 10–19 days, 20–29 days, or all 30 days	Yes: 1 or 2 days, 3–5 days, 6–9 days, 10–19 days, 20–29 days, or all 30 days No: 0 days
Marijuana	During the past 30 days, how many times did you use marijuana?	0 times, 1 or 2 times, 3–9 times, 10–19 times, 20–39 times, or ≥40 times	Yes: 1 or 2 times, 3–9 times, 10–19 times, 20–39 times, or ≥40 times No: 0 times
Prescription opioid misuse*	During the past 30 days, how many times did you take prescription pain medicine without a doctor’s prescription or differently than how a doctor told you to use it?	0 times, 1 or 2 times, 3–9 times, 10–19 times, 20–39 times, or ≥40 times	Yes: 1 or 2 times, 3–9 times, 10–19 times, 20–39 times, or ≥40 times No: 0 times
**Lifetime use**			
Electronic vapor products	Have you ever used an electronic vapor product?	Yes or no	Yes: Yes No: No
Marijuana	During your life, how many times have you used marijuana?	0 times, 1 or 2 times, 3–9 times, 10–19 times, 20–39 times, 40–99 times, or ≥100 times	Yes: 1 or 2 times, 3–9 times, 10–19 times, 20–39 times, 40–99 times, or ≥100 times No: 0 times
Prescription opioid misuse*	During your life, how many times have you taken prescription pain medicine without a doctor's prescription or differently than how a doctor told you to use it?	0 times, 1 or 2 times, 3–9 times, 10–19 times, 20–39 times, or ≥40 times	Yes: 1 or 2 times, 3–9 times, 10–19 times, 20–39 times, or ≥40 times No: 0 times
Cocaine	During your life, how many times have you used any form of cocaine, including powder, crack, or freebase?	0 times, 1 or 2 times, 3–9 times, 10–19 times, 20–39 times, or ≥40 times	Yes: 1 or 2 times, 3–9 times, 10–19 times, 20–39 times, or ≥40 times No: 0 times
Inhalants	During your life, how many times have you sniffed glue, breathed the contents of aerosol spray cans, or inhaled any paints or sprays to get high?	0 times, 1 or 2 times, 3–9 times, 10–19 times, 20–39 times, or ≥40 times	Yes: 1 or 2 times, 3–9 times, 10–19 times, 20–39 times, or ≥40 times No: 0 times
Heroin	During your life, how many times have you used heroin (also called smack, junk, or China White)?	0 times, 1 or 2 times, 3–9 times, 10–19 times, 20–39 times, or ≥40 times	Yes: 1 or 2 times, 3–9 times, 10–19 times, 20–39 times, or ≥40 times No: 0 times
Methamphetamines	During your life, how many times have you used methamphetamines (also called speed, crystal meth, crank, ice, or meth)?	0 times, 1 or 2 times, 3–9 times, 10–19 times, 20–39 times, or ≥40 times	Yes: 1 or 2 times, 3–9 times, 10–19 times, 20–39 times, or ≥40 times No: 0 times
Ecstasy	During your life, how many times have you used ecstasy (also called MDMA or Molly)?	0 times, 1 or 2 times, 3–9 times, 10–19 times, 20–39 times, or ≥40 times	Yes: 1 or 2 times, 3–9 times, 10–19 times, 20–39 times, or ≥40 times No: 0 times
Hallucinogenic drugs	During your life, how many times have you used hallucinogenic drugs, such as LSD, acid, PCP, angel dust, mescaline, or mushrooms?	0 times, 1 or 2 times, 3–9 times, 10–19 times, 20–39 times, or ≥40 times	Yes: 1 or 2 times, 3–9 times, 10–19 times, 20–39 times, or ≥40 times No: 0 times
Selected illicit drugs	During your life, how many times have you • used any form of cocaine, including powder, crack, or freebase? • sniffed glue, breathed the contents of aerosol spray cans, or inhaled any paints or sprays to get high? • used heroin (also called smack, junk, or China White)? • used methamphetamines (also called speed, crystal meth, crank, ice, or meth)? • used ecstasy (also called MDMA or Molly)? • used hallucinogenic drugs, such as LSD, acid, PCP, angel dust, mescaline, or mushrooms?	0 times, 1 or 2 times, 3–9 times, 10–19 times, 20–39 times, or ≥40 times	Yes: 1 or 2 times, 3–9 times, 10–19 times, 20–39 times, or ≥40 times for any included substance No: 0 times for all included substances
Injection of illegal drug	During your life, how many times have you used a needle to inject any illegal drug into your body?	0 times, 1 time, or ≥2 times	Yes: 1 time or ≥2 times No: 0 times

* Questions on current and lifetime prescription opioid misuse are preceded with the following description, “The next 2 questions ask about the use of prescription pain medicine without a doctor's prescription or differently than how a doctor told you to use it. For these questions, count drugs such as codeine, Vicodin, OxyContin, hydrocodone, and Percocet.”

Demographic variables included sex (female or male), race and ethnicity (American Indian or Alaska Native [AI/AN], Asian, Black or African American [Black], Hispanic or Latino [Hispanic], Middle Eastern or North African [ME/NA], Native Hawaiian or Pacific Islander, White, or multiracial [selected more than one racial category]), and grade (9, 10, 11, or 12). Persons of Hispanic origin might be of any race but are categorized as Hispanic; all racial groups are non-Hispanic.

The percent of missing responses for substance use behaviors varied by survey item, ranging from 1.6% (current cigarette use) to 37.3% (lifetime use of selected illicit drugs), with 12 of 18 behaviors having <15% missing data. To reduce respondent effort, students in schools selected for both the national and site-level YRBS samples were administered only one questionnaire: either the national or site-specific questionnaire. Because site-specific questionnaires might not include all questions on the national YRBS questionnaire, some students included in the national sample were not asked certain substance use questions. These procedures contributed to the higher levels of missing responses for some measures. Demographic variables had low levels of missing responses, with the percent of missing responses ranging from 0.4% (sex and grade) to 0.9% (race and ethnicity). Estimates for each substance use behavior were calculated among students with valid responses to that specific behavior; therefore, analytic denominators varied across substance use behaviors. 

School-level urban-rural classification was based on the NCES locale classification scheme, which describes the type of area in which a school is located based on population size or proximity to populated areas (https://nces.ed.gov/programs/edge/Geographic/LocaleBoundaries). Additional technical details about the NCES locale framework are available (https://nces.ed.gov/programs/edge/docs/EDGE_LOCALE_TECHDOC.pdf). School-level urban-rural classification included three levels: city, suburb or town, and rural. Urban locale subtypes included city, suburbs, and town. NCES locale linkage was not conducted for students who attended private schools (approximately 5%); therefore, findings on urban-rural differences in substance use behaviors are only representative of U.S. public high school students.

### Analysis

Weighted prevalence and 95% CIs for substance use behaviors were calculated overall and by demographic characteristics and school urban-rural classification. Prevalence estimates with a denominator <30 were considered statistically unreliable and suppressed (*6*); estimates for Native Hawaiian or Pacific Islander students are not presented because the denominator was <30 for several substance use behaviors. Differences in prevalence of substance use behaviors by demographic characteristics and urban-rural classification were assessed using design-adjusted *t* tests for pairwise comparisons. Logistic regression analyses were used to examine associations between urban-rural classification and substance use behaviors. Because the demographic composition of students might differ across urban-rural classifications and these characteristics might also be associated with substance use, adjusted prevalence ratios (aPRs) were estimated using predicted marginals from models adjusted for sex, race and ethnicity, and grade. All estimates were calculated using Taylor series linearization. Pairwise *t* tests were considered statistically significant at p<0.05, and prevalence ratios were considered statistically significant when the 95% CIs did not cross the null hypothesis value of 1.0. Given the descriptive and hypothesis-generating nature of this study, adjustments were not made for multiple comparisons. Each statistical test addressed a distinct research question and was interpreted independently. All analyses were conducted using SAS-callable SUDAAN (version 11.0.4; RTI International) to account for the complex sampling design and weighting.

## Results

Among U.S. high school students in 2025, the most commonly reported substance use behaviors were lifetime use of electronic vapor products (30.2%) and marijuana (25.5%) and current use of alcohol (20.3%) and electronic vapor products (16.0%) (Table 2).

**TABLE 2:** Prevalence of current and lifetime use of specific substances among high school students, by demographic characteristics — Youth Risk Behavior Survey, United States, 2025*

Variable	Overall	Sex		Race and ethnicity^†,§^							Grade			
		Female	Male	AI/AN	Asian	Black or African American	Hispanic or Latino	ME/NA	White	Multiracial	9	10	11	12
		% (95% CI)	% (95% CI)	% (95% CI)	% (95% CI)	% (95% CI)	% (95% CI)	% (95% CI)	% (95% CI)	% (95% CI)	% (95% CI)	% (95% CI)	% (95% CI)	% (95% CI)
**Current use** ^¶^														
Cigarettes**	**3.5 (2.7–4.5)**	2.7 (1.8–4.1)	4.2 (3.4–5.1)	8.5 (3.1–20.9)	2.1 (1.1–4.1)	1.4 (0.9–2.1)	3.4 (2.6–4.6)	6.1 (1.8–18.6)	4.2 (3.0–6.0)	2.6 (1.3–5.3)	2.1 (1.1–3.8)	2.8 (1.9–4.2)	4.2 (2.9–5.9)	5.1 (4.0–6.5)
Cigars^††^	**3.2 (2.6–3.9)**	1.8 (1.2–2.7)	4.5 (3.8–5.3)	13.0 (3.4–38.8)	1.0 (0.4–2.5)	4.8 (3.1–7.3)	3.3 (2.5–4.4)	7.7 (2.5–21.3)	2.8 (2.1–3.8)	2.7 (1.4–5.1)	2.1 (1.4–3.2)	3.0 (2.1–4.3)	3.5 (2.5–4.9)	4.2 (2.9–6.0)
Smokeless tobacco^§§^	**4.1 (3.0–5.5)**	1.8 (1.3–2.4)	6.2 (4.5–8.7)	5.5 (1.1–24.3)	3.4 (1.6–6.8)	2.0 (1.2–3.2)	2.8 (1.8–4.3)	6.9 (2.1–19.8)	5.4 (3.8–7.5)	2.3 (1.3–4.0)	3.3 (1.9–5.7)	3.5 (2.3–5.5)	4.8 (3.4–6.7)	4.6 (3.3–6.5)
Electronic vapor products^¶¶^	**16.0 (14.3–17.9)**	18.0 (15.9–20.4)	14.2 (12.5–16.1)	19.0 (11.8–29.3)	4.7 (2.8–7.7)	16.7 (14.6–19.2)	16.4 (13.7–19.5)	6.1 (2.8–12.6)	16.9 (14.4–19.8)	17.5 (13.8–22.0)	11.1 (9.0–13.7)	16.0 (13.1–19.2)	18.3 (14.9–22.3)	19.5 (16.5–22.9)
Alcohol***	**20.3 (18.1–22.6)**	20.9 (18.2–23.9)	19.8 (17.1–22.7)	24.8 (15.1–37.9)	12.7 (9.0–17.7)	10.7 (9.1–12.5)	18.8 (16.6–21.2)	19.9 (10.2–35.0)	23.7 (20.4–27.3)	24.1 (18.0–31.4)	11.5 (9.3–14.1)	16.8 (14.1–19.7)	23.5 (19.4–28.2)	30.9 (27.4–34.7)
Marijuana⁠^†⁠†⁠†^	**12.9 (11.2–14.8)**	13.4 (11.5–15.6)	12.5 (10.6–14.7)	12.1 (5.9–23.3)	2.9 (1.6–5.4)	15.4 (12.7–18.5)	12.7 (10.4–15.3)	12.7 (5.8–25.6)	13.4 (11.0–16.3)	13.4 (10.1–17.6)	7.4 (5.9–9.4)	11.9 (9.9–14.3)	15.2 (11.5–19.8)	18.1 (15.2–21.5)
Prescription opioid misuse^§⁠§⁠§^	**5.0 (4.3–5.8)**	6.0 (4.8–7.4)	4.1 (3.3–5.2)	4.3 (1.8–10.1)	7.2 (4.9–10.5)	7.5 (6.2–9.0)	6.4 (5.2–8.0)	4.0 (1.5–9.9)	3.6 (2.8–4.5)	4.5 (2.6–7.9)	5.6 (4.3–7.2)	4.6 (3.3–6.3)	5.2 (4.2–6.5)	4.5 (3.4–5.9)
**Lifetime use**														
Electronic vapor products^¶⁠¶⁠¶^	**30.2 (27.5–33.1)**	33.3 (30.3–36.4)	27.4 (24.5–30.4)	32.1 (18.9–48.9)	11.7 (8.7–15.6)	31.3 (27.3–35.5)	33.6 (30.1–37.2)	21.2 (15.1–29.1)	30.1 (26.6–33.9)	30.5 (25.1–36.5)	23.5 (19.6–28.0)	28.6 (25.0–32.5)	33.1 (29.1–37.4)	36.8 (33.3–40.4)
Marijuana⁠*⁠*⁠*⁠*	**25.5 (22.5–28.7)**	27.4 (23.8–31.3)	23.8 (20.9–27.0)	30.9 (17.2–49.0)	6.8 (4.1–11.1)	25.7 (22.1–29.7)	29.1 (25.7–32.7)	23.5 (9.8–46.7)	25.4 (21.8–29.5)	26.7 (20.0–34.7)	14.7 (12.0–17.8)	23.4 (20.0–27.2)	29.7 (24.4–35.6)	36.3 (32.3–40.4)
Prescription opioid misuse^†⁠†⁠†⁠†^	**10.6 (9.5–11.9)**	12.3 (10.5–14.4)	9.1 (7.9–10.4)	12.8 (4.6–30.7)	10.9 (8.2–14.3)	13.4 (11.6–15.4)	11.9 (9.8–14.3)	8.7 (3.5–20.0)	9.5 (8.1–11.0)	10.5 (7.8–13.9)	11.0 (9.1–13.2)	10.9 (9.0–13.2)	11.1 (9.7–12.7)	9.4 (7.8–11.4)
Cocaine^§⁠§⁠§⁠§⁠^	**2.0 (1.4–2.8)**	1.5 (0.9–2.3)	2.5 (1.7–3.6)	2.5 (1.0–6.5)	1.7 (0.7–4.1)	2.5 (1.5–4.0)	2.4 (1.5–3.9)	4.0 (1.5–10.2)	1.6 (1.0–2.7)	1.9 (0.9–3.9)	2.3 (1.2–4.5)	1.5 (0.9–2.3)	1.8 (1.2–2.7)	2.2 (1.3–3.7)
Inhalants⁠^¶⁠¶⁠¶⁠¶^	**5.0 (4.2–5.9)**	5.1 (3.9–6.5)	5.0 (4.1–6.0)	8.5 (2.2–27.3)	3.6 (1.8–7.1)	5.6 (4.0–7.9)	5.0 (3.7–6.7)	6.6 (1.7–22.8)	5.0 (4.0–6.3)	4.9 (2.4–9.9)	6.9 (5.6–8.5)	4.5 (3.4–5.9)	4.6 (3.3–6.4)	3.5 (2.4–5.0)
Heroin*⁠*⁠*⁠*⁠*	**1.1 (0.8–1.4)**	0.7 (0.4–1.0)	1.5 (1.0–2.1)	0.7 (0.1–4.1)	1.0 (0.3–2.7)	1.9 (1.1–3.3)	1.2 (0.8–1.7)	5.3 (2.0–13.2)	0.7 (0.4–1.2)	0.8 (0.3–2.2)	1.4 (0.7–2.8)	0.8 (0.5–1.3)	1.1 (0.7–1.8)	0.7 (0.4–1.2)
Methamphetamines^†⁠†⁠†⁠†⁠†^	**1.3 (0.9–2.0)**	1.1 (0.7–1.8)	1.5 (1.0–2.2)	0.1 (0.0–1.2)	1.0 (0.4–2.8)	1.8 (1.0–3.1)	1.4 (1.0–2.0)	4.4 (1.4–12.9)	1.1 (0.6–2.1)	0.9 (0.4–2.5)	1.8 (0.8–3.7)	0.9 (0.5–1.4)	1.4 (0.9–2.3)	1.0 (0.7–1.6)
Ecstasy^§§§§§^	**1.8 (1.3–2.6)**	1.5 (0.9–2.4)	2.1 (1.5–3.0)	1.2 (0.2–5.5)	0.8 (0.3–2.7)	2.4 (1.4–3.9)	2.2 (1.6–3.0)	5.7 (1.5–18.7)	1.5 (0.8–2.5)	1.7 (0.8–3.7)	1.8 (0.8–3.8)	1.3 (0.9–2.0)	1.8 (1.1–2.8)	2.3 (1.6–3.4)
Hallucinogenic drugs⁠^¶⁠¶⁠¶⁠¶⁠¶^	**5.2 (4.1–6.6)**	4.1 (2.7–6.2)	6.3 (5.2–7.7)	2.1 (0.7–6.2)	2.9 (1.1–7.2)	3.4 (2.1–5.5)	6.8 (5.1–9.1)	3.3 (1.1–9.3)	5.0 (3.5–7.1)	5.4 (2.9–10.1)	3.1 (2.2–4.2)	5.3 (3.7–7.7)	4.8 (3.4–6.8)	8.1 (6.0–10.8)
Selected illicit drugs*⁠*⁠*⁠*⁠*⁠*	**9.7 (8.3–11.3)**	9.1 (7.1–11.6)	10.3 (8.8–12.0)	10.9 (3.5–29.4)	6.8 (3.4–12.9)	9.1 (6.6–12.6)	11.3 (9.1–14.0)	6.6 (2.1–18.9)	9.2 (7.3–11.5)	10.0 (6.0–16.4)	9.5 (7.7–11.6)	8.6 (6.7–11.1)	9.7 (7.6–12.4)	11.0 (8.5–14.2)
Injection of illegal drug^††††††^	**1.0 (0.7–1.6)**	0.4 (0.2–0.8)	1.6 (1.0–2.6)	0.7 (0.1–4.1)	0.9 (0.3–2.8)	1.8 (1.0–3.1)	1.2 (0.8–2.1)	3.9 (1.2–12.3)	0.6 (0.3–1.1)	1.6 (0.5–5.3)	1.8 (0.9–3.5)	0.6 (0.4–1.0)	0.8 (0.4–1.4)	0.8 (0.5–1.3)

**Abbreviations:** AI/AN = American Indian or Alaska Native; ME/NA = Middle Eastern or North African.

* N = 10,710. The total number (N) of students answering each question varied. Data might be missing because 1) students in schools selected for both national and site-specific YRBS samples completed only one questionnaire; therefore, when the site-specific questionnaire was administered, it might not have included all questions on the national YRBS; 2) the student did not answer the question; or 3) the response was set to missing because of an out-of-range response or logical inconsistency. Prevalence estimates in each category are calculated based on nonmissing data.

^†^ Persons of Hispanic or Latino (Hispanic) origin might be of any race but are categorized as Hispanic; all racial groups are non-Hispanic.

^§^ Prevalence estimates with a denominator <30 were considered statistically unreliable and therefore were suppressed; estimates for Native Hawaiian or Pacific Islander students are not presented because the denominator was <30 for several substance use behaviors.

^¶^ Previous 30 days before the survey.

** As determined by *t* test analysis with Taylor series linearization (p<0.05), female students significantly differed from male students, Asian students significantly differed from White students, Black or African American (Black) students significantly differed from Hispanic and White students, grade 9 students significantly differed from grade 11 and 12 students, and grade 10 students significantly differed from grade 12 students.

^††^ As determined by *t* test analysis with Taylor series linearization (p<0.05), female students significantly differed from male students; Asian students significantly differed from Black, Hispanic, and White students; Black students significantly differed from White and multiracial students; and grade 9 students significantly differed from grade 12 students.

^§§^ As determined by *t* test analysis with Taylor series linearization (p<0.05), female students significantly differed from male students, and White students significantly differed from Black, Hispanic, and multiracial students.

^¶¶^ As determined by *t* test analysis with Taylor series linearization (p<0.05), female students significantly differed from male students; Asian students significantly differed from AI/AN, Black, Hispanic, White, and multiracial students; ME/NA students significantly differed from AI/AN, Black, Hispanic, White, and multiracial students; grade 9 students significantly differed from grade 10, 11, and 12 students; and grade 10 students significantly differed from grade 12 students.

*** As determined by *t* test analysis with Taylor series linearization (p<0.05), Asian students significantly differed from AI/AN, Hispanic, White, and multiracial students; Black students significantly differed from AI/AN, Hispanic, White, and multiracial students; Hispanic students significantly differed from White students; grade 9 students significantly differed from grade 10, 11, and 12 students; grade 10 students significantly differed from grade 11 and 12 students; and grade 11 students significantly differed from grade 12 students.

^†††^ As determined by *t* test analysis with Taylor series linearization (p<0.05), Asian students significantly differed from AI/AN, Black, Hispanic, ME/NA, White, and multiracial students; grade 9 students significantly differed from grade 10, 11, and 12 students; and grade 10 students significantly differed from grade 12 students.

^§§§^ As determined by *t* test analysis with Taylor series linearization (p<0.05), female students significantly differed from male students; Asian students significantly differed from ME/NA, White, and multiracial students; Black students significantly differed from White and multiracial students; and Hispanic students significantly differed from White students.

^¶¶¶^ As determined by *t* test analysis with Taylor series linearization (p<0.05), female students significantly differed from male students; Asian students significantly differed from AI/AN, Black, Hispanic, ME/NA, White, and multiracial students; ME/NA students significantly differed from Black, Hispanic, and White students; grade 9 students significantly differed from grade 10, 11, and 12 students; and grade 10 students significantly differed from grade 11 and 12 students.

**** As determined by *t* test analysis with Taylor series linearization (p<0.05), female students significantly differed from male students; Asian students significantly differed from AI/AN, Black, Hispanic, White, and multiracial students; grade 9 students significantly differed from grade 10, 11, and 12 students; grade 10 students significantly differed from grade 11 and 12 students; and grade 11 students significantly differed from grade 12 students.

^††††^ As determined by *t* test analysis with Taylor series linearization (p<0.05), female students significantly differed from male students, and Black students significantly differed from White students.

^§§§§^ As determined by *t* test analysis with Taylor series linearization (p<0.05), female students significantly differed from male students.

^¶¶¶¶^ As determined by *t* test analysis with Taylor series linearization (p<0.05), grade 9 students significantly differed from grade 10, 11, and 12 students.

***** As determined by *t* test analysis with Taylor series linearization (p<0.05), female students significantly differed from male students, and White students significantly differed from Black and Hispanic students.

^†††††^ As determined by *t* test analysis with Taylor series linearization (p<0.05), AI/AN students significantly differed from Black, Hispanic, and White students.

^§§§§§^ As determined by *t* test analysis with Taylor series linearization (p<0.05), Hispanic students significantly differed from Asian and White students.

^¶¶¶¶¶^ As determined by *t* test analysis with Taylor series linearization (p<0.05), female students significantly differed from male students; Hispanic students significantly differed from AI/AN, Asian, and Black students; grade 9 students significantly differed from grade 10, 11, and 12 students; and grade 11 students significantly differed from grade 12 students.

****** No statistically significant differences were observed.

^††††††^ As determined by *t* test analysis with Taylor series linearization (p<0.05), female students significantly differed from male students, and White students significantly differed from Black and Hispanic students.

Substance use behaviors varied by sex, race and ethnicity, and grade. Male students had a higher prevalence than female students of current use of cigarettes (4.2% versus 2.7%), cigars (4.5% versus 1.8%), and smokeless tobacco (6.2% versus 1.8%), as well as lifetime use of cocaine (2.5% versus 1.5%), heroin (1.5% versus 0.7%), hallucinogenic drugs (6.3% versus 4.1%), and injection of illegal drugs (1.6% versus 0.4%). In contrast, female students had a higher prevalence than male students of current use of electronic vapor products (18.0% versus 14.2%) and prescription opioid misuse (6.0% versus 4.1%), as well as lifetime electronic vapor product use (33.3% versus 27.4%), marijuana use (27.4% versus 23.8%), and prescription opioid misuse (12.3% versus 9.1%). Differences by race and ethnicity were identified for 15 of 18 substance use behaviors. For nine substance use behaviors, Asian students had a lower prevalence than at least one other racial or ethnic group. For example, Asian students had a lower prevalence of current use of electronic vapor products (4.7%) than AI/AN (19.0%), multiracial (17.5%), White (16.9%), Black (16.7%), and Hispanic (16.4%) students. AI/AN and Hispanic students had a higher prevalence than at least one other racial or ethnic group for five and 13 substance use behaviors, respectively. Among Black, White, multiracial, and ME/NA students, race and ethnicity patterns varied by substance use behavior. Nine of 18 substance use behaviors varied by grade, with substance use behaviors being more common among students in higher grade levels for most behaviors.

Five substance use behaviors varied by urban-rural classification among public high school students (Table 3). Students in rural public schools consistently reported a higher prevalence of tobacco use and electronic vapor product use than students in city and suburb or town public schools. Compared with students in city public schools, students in rural public schools had a higher prevalence of current cigarette (5.8% versus 3.0%), smokeless tobacco (6.4% versus 2.8%), and electronic vapor product use (21.7% versus 15.3%), as well as lifetime electronic vapor product use (38.9% versus 29.3%). Students in rural public schools also had a higher prevalence than students in suburb or town public schools of current use of cigarettes (5.8% versus 3.2%), cigars (4.4% versus 3.1%), smokeless tobacco (6.4% versus 3.1%), and electronic vapor products (21.7% versus 15.2%), as well as lifetime use of electronic vapor products (38.9% versus 28.5%).

**TABLE 3:** Prevalence of current and lifetime use of specific substances among public high school students, by urban-rural classification — Youth Risk Behavior Survey, United States, 2025*

Variable	School-level urban-rural classification^†^		
	City	Suburb or town	Rural
	% (95% CI)	% (95% CI)	% (95% CI)
**Current use** ^§^			
Cigarettes	3.0 (1.9–4.8)^¶^	3.2 (2.3–4.3)**	5.8 (4.0–8.2)
Cigars	2.8 (1.7–4.3)	3.1 (2.4–4.2)**	4.4 (3.2–6.0)
Smokeless tobacco	2.8 (1.8–4.4)^¶^	3.1 (2.3–4.2)**	6.4 (4.1–9.8)
Electronic vapor products	15.3 (12.9–18.1)^¶^	15.2 (12.7–18.0)**	21.7 (18.3–25.5)
Alcohol	18.8 (16.0–22.0)	18.6 (16.0–21.7)	23.2 (18.3–29.0)
Marijuana	13.0 (10.3–16.4)	12.9 (10.7–15.6)	14.3 (10.3–19.5)
Prescription opioid misuse	5.6 (4.1–7.6)	5.0 (4.1–6.2)	5.2 (3.8–7.0)
**Lifetime use**			
Electronic vapor products	29.3 (25.2–33.7)^¶^	28.5 (25.1–32.1)**	38.9 (33.7–44.3)
Marijuana	25.5 (21.2–30.3)	25.9 (21.4–30.9)	25.5 (21.5–30.0)
Prescription opioid misuse	10.6 (8.9–12.7)	10.9 (9.2–12.9)	11.9 (9.6–14.6)
Cocaine	2.5 (1.6–3.8)	2.0 (1.3–3.0)	1.9 (0.9–4.4)
Inhalants	4.2 (2.9–6.1)	5.0 (4.0–6.1)	5.4 (4.0–7.2)
Heroin	1.1 (0.8–1.7)	1.0 (0.7–1.4)	1.4 (0.6–2.9)
Methamphetamines	1.3 (0.8–2.3)	1.4 (0.8–2.2)	1.5 (0.6–3.4)
Ecstasy	2.3 (1.3–3.9)	1.6 (1.1–2.3)	2.0 (0.8–5.0)
Hallucinogenic drugs	6.1 (3.9–9.3)	5.2 (3.9–6.9)	4.8 (2.6–8.4)
Selected illicit drugs	9.9 (7.0–13.8)	9.6 (7.9–11.8)	9.1 (7.0–11.8)
Injection of illegal drug	1.3 (0.7–2.1)	1.0 (0.5–1.9)	1.0 (0.4–2.4)

* N = 10,161. Data on urban-rural classification was missing for 549 students. School-level urban-rural classification included three levels: city (n = 4,367), suburb or town (n = 4,461), and rural (n = 1,333). The total number of students answering each question varied. Data might be missing because 1) students in schools selected for both national and site-specific YRBS samples completed only one questionnaire; therefore, when the site-specific questionnaire was administered, it might not have included all questions on the national YRBS; 2) the student did not answer the question; or 3) the response was set to missing because of an out-of-range response or logical inconsistency. Prevalence estimates in each category are calculated based on nonmissing data.

^†^ School urban-rural classification was based on the National Center for Education Statistics locale classification scheme. The three-level urban-rural classification scheme comprised city, a combined suburb and town category, and rural. https://nces.ed.gov/programs/edge/Geographic/LocaleBoundaries

^§^ Previous 30 days before the survey.

^¶^ Students in city schools significantly differed from students in rural schools, as determined by *t* test analysis with Taylor series linearization (p<0.05).

** Students in suburb or town schools significantly differed from students in rural schools, as determined by *t* test analysis with Taylor series linearization (p<0.05).

In analyses adjusted for sex, race and ethnicity, and grade, students in rural public schools compared with city public schools had a higher prevalence of current use of smokeless tobacco (aPR = 1.84; 95% CI = 1.08–3.13) and electronic vapor products (aPR = 1.37; 95% CI = 1.08–1.74), as well as lifetime use of electronic vapor products (aPR = 1.34; 95% CI = 1.11–1.62) (Table 4). Students in rural public schools compared with suburb or town public schools also had a higher prevalence of current use of cigarettes (aPR = 1.62; 95% CI = 1.04–2.50), cigars (aPR = 1.42; 95% CI = 1.04–1.92), smokeless tobacco (aPR = 1.74; 95% CI = 1.10–2.74), and electronic vapor products (aPR = 1.40; 95% CI = 1.13–1.73), as well as lifetime use of electronic vapor products (aPR = 1.37; 95% CI = 1.17–1.59).

**TABLE 4:** Associations between school-level urban-rural classification and current and lifetime use of specific substances among public high school students — Youth Risk Behavior Survey, United States, 2025*

Variable	School-level urban-rural classification^†^		
	Rural versus city	Rural versus suburb or town	City versus suburb or town
	aPR^§^ (95% CI)	aPR^§^ (95% CI)	aPR^§^ (95% CI)
**Current use** ^¶^			
Cigarettes	1.71 (0.92–3.17)	1.62 (1.04–2.50)**	0.95 (0.57–1.57)
Cigars	1.61 (0.91–2.84)	1.42 (1.04–1.92)**	0.88 (0.49–1.56)
Smokeless tobacco	1.84 (1.08–3.13)**	1.74 (1.10–2.74)**	0.94 (0.68–1.31)
Electronic vapor products	1.37 (1.08–1.74)**	1.40 (1.13–1.73)**	1.02 (0.80–1.30)
Alcohol	1.15 (0.89–1.49)	1.14 (0.88–1.47)	0.99 (0.84–1.17)
Marijuana	1.05 (0.69–1.60)	1.06 (0.78–1.45)	1.01 (0.76–1.35)
Prescription opioid misuse	1.14 (0.72–1.80)	1.16 (0.81–1.65)	1.02 (0.69–1.50)
**Lifetime use**			
Electronic vapor products	1.34 (1.11–1.62)**	1.37 (1.17–1.59)**	1.02 (0.86–1.21)
Marijuana	0.99 (0.78–1.26)	0.97 (0.78–1.22)	0.98 (0.79–1.22)
Prescription opioid misuse	1.23 (0.93–1.63)	1.16 (0.90–1.49)	0.94 (0.74–1.18)
Cocaine	0.84 (0.35–2.02)	0.99 (0.41–2.36)	1.18 (0.75–1.86)
Inhalants	1.21 (0.71–2.08)	1.04 (0.72–1.50)	0.86 (0.56–1.31)
Heroin	1.41 (0.64–3.10)	1.39 (0.64–3.05)	0.99 (0.65–1.51)
Methamphetamines	1.24 (0.48–3.20)	1.13 (0.48–2.67)	0.91 (0.60–1.37)
Ecstasy	0.98 (0.33–2.92)	1.25 (0.47–3.30)	1.27 (0.82–1.97)
Hallucinogenic drugs	0.75 (0.38–1.50)	0.88 (0.47–1.64)	1.17 (0.73–1.87)
Selected illicit drugs	0.93 (0.61–1.43)	0.94 (0.70–1.27)	1.01 (0.71–1.45)
Injection of illegal drug	1.00 (0.42–2.41)	1.18 (0.43–3.23)	1.18 (0.68–2.04)

**Abbreviation:** aPR = adjusted prevalence ratio.

* N = 10,161. Data on urban-rural classification was missing for 549 students. School-level urban-rural classification included three levels: city (n = 4,367), suburb or town (n = 4,461), and rural (n = 1,333). The total number of students answering each question varied. Data might be missing because 1) students in schools selected for both national and site-specific YRBS samples completed only one questionnaire; therefore, when the site-specific questionnaire was administered, it might not have included all questions on the national YRBS; 2) the student did not answer the question; or 3) the response was set to missing because of an out-of-range response or logical inconsistency. Analyses for each behavior were based on nonmissing data.

^†^ School urban-rural classification was based on the National Center for Education Statistics locale classification scheme. The three-level urban-rural classification scheme comprised city, a combined suburb and town category, and rural. https://nces.ed.gov/programs/edge/Geographic/LocaleBoundaries

^§^ Adjusted for sex, race and ethnicity, and grade.

^¶^ Previous 30 days before the survey.

** Statistically significant; 95% CIs do not cross the null hypothesis value of 1.0.

## Discussion

From 2013 to 2023, high school students’ use of several substances, including alcohol, marijuana, prescription opioids not prescribed to them, and other illicit drugs, declined (*4*). However, data from the 2025 YRBS indicate that adolescent substance use remains a substantial public health concern, as use of certain substances remains prevalent. Nearly one in three high school students reported lifetime use of electronic vapor products, and approximately one in four reported lifetime marijuana use, making these the most commonly reported lifetime substance use behaviors in this survey cycle. Current alcohol and electronic vapor product use were also commonly reported behaviors, suggesting that a substantial proportion of adolescents are actively engaging in substance use during a critical period of physical and neurologic development (*3*).

This report uses data from the national YRBS to describe urban-rural differences in a range of substance use behaviors. Students attending rural public schools had a higher prevalence of use of several tobacco-related products, including current use of cigarettes, cigars, smokeless tobacco, and electronic vapor products, as well as lifetime use of electronic vapor products. Differences observed in patterns of current and lifetime use might reflect variations in initiation, continued use, and cessation by urban-rural classification, as current use captures recent substance use behaviors, whereas lifetime use captures any past use, including experimentation. In addition, unadjusted and adjusted findings related to urban-rural differences were largely consistent. However, the difference in current cigarette use between students in rural and city public schools was not statistically significant after adjusting for sex, race and ethnicity, and grade, suggesting that observed differences in the demographic composition of students across rural and city schools might partly explain the unadjusted association. These findings are consistent with findings from the National Youth Tobacco Survey, which indicated that rural high school students are more likely to smoke cigarettes and use smokeless tobacco than urban students (*7*). Similarly, findings from the Monitoring the Future survey also demonstrated that high school students in rural settings were more likely to report use of several tobacco-related products than students in city or suburban settings (*8*).

Multiple contextual factors might contribute to these observed differences. Social norms and community-level exposure to tobacco use might influence patterns of use (*7*). Rural communities often have reduced access to substance use prevention and treatment services, with barriers including longer travel distances and limited provider availability (*1*). Reducing tobacco use among adolescents likely requires expanding access to evidence-based tobacco prevention interventions. Schools are essential in these efforts, as they are both a venue where substances are accessed and an ideal setting for the provision of evidence-based prevention programs and services. These efforts might be particularly important in rural schools, because some rural communities experience a shortage of primary care and mental health providers and services (https://data.hrsa.gov/topics/health-workforce/shortage-areas).

In contrast, no statistically significant differences by urban-rural classification were observed for alcohol, marijuana, and selected illicit drug use, consistent with data from the National Survey on Drug Use and Health showing generally comparable prevalence estimates for most past-year illicit drug use between metropolitan and nonmetropolitan youths aged 12–17 years (*2*). Collectively, these findings suggest that urban-rural differences in adolescent substance use might be concentrated in tobacco-related behaviors rather than observed across all substances. One possible explanation is that alcohol, marijuana, and other drug use among adolescents might be more closely related to risk and protective factors that span both urban and rural settings, such as peer substance use, perceived risk, parental monitoring, school connectedness, and the broader environment, than to rurality alone (*1*,*3*,*9*). Previous research suggests that urban-rural differences in tobacco-related product use might be associated with tobacco norms, product availability, and local tobacco control environments (*7*).

In addition to observed urban-rural differences, substance use behaviors differed by demographic characteristics, highlighting potential opportunities for evidence-based prevention strategies tailored to the needs of different adolescent populations and communities. Males reported higher prevalence of cigarette, cigar, and smokeless tobacco use than females. Females reported higher prevalence of electronic vapor product use than males, consistent with previously reported changes in sex-specific patterns of youth electronic cigarette use (*10*).

Observed racial and ethnic differences in substance use varied based on the specific substance used. For example, use of several substances was less common among Asian students. A previous study had a similar finding of lower marijuana use among Asian adolescents, which has been attributed to factors such as higher perceived risk and greater disapproval from parents, peers, and close friends (*11*). Although higher alcohol use was observed among AI/AN students compared with certain racial and ethnic groups, the factors underlying these differences were not examined in this study. Previous research has identified broader societal and contextual factors that might influence substance use risk, including access to prevention and treatment services and community norms (*12*). Several substance use behaviors were more common among students in higher grade levels, suggesting potential value in early, developmentally appropriate prevention programming that begins before initiation of use. However, grade-related patterns were not consistent across all substances, which might reflect variations in the popularity or availability of specific substances among different grades.

Schools might be well positioned to address adolescent substance use. A 2019 study found that young people in states with requirements for alcohol use prevention programs in high schools had lower risk for alcohol dependence and abuse than those in states with no requirement (*13*). Characteristics of effective school-based substance use prevention programs for older adolescents might include those focused on building skills such as refusal techniques, self-control, and decision-making, as well as programs aligning instruction with adolescents’ individual future goals (*14*).

CDC, in collaboration with the CDC Foundation and the Conrad Hilton Foundation, developed and implemented the Teens Linked to Care pilot program, a school-based framework to address substance use and related risky behaviors among youths in rural communities involving health education, primary prevention, and referrals to treatment and services (https://www.cdc.gov/youth-behavior/risk-behaviors/teens-linked-to-care.html).

CDC also supports local education agencies to implement evidence-based strategies to strengthen health education, increase access to health services, and foster safe and supportive school environments. The program has been associated with significant decreases in multiple adolescent health risk behaviors, including reductions in marijuana use (*15*). Additionally, the Drug-Free Communities (DFC) Support Program, administered by the Office of National Drug Control Policy and managed through a partnership with CDC, supports multisector community coalitions, consisting of members from schools, businesses, parents, law enforcement, and community organizations, and works to increase local capacity to prevent and reduce youth substance use. DFC was associated with decreases in past 30-day prescription drug misuse and use of alcohol, tobacco, and marijuana and among middle school–aged and high school–aged youths when comparing their first and most recent report (https://www.cdc.gov/overdose-prevention/php/drug-free-communities/program-success.html). Although multiple factors might have influenced these decreases, the findings suggest potential value in long-term community-based youth substance use prevention efforts.

Finally, school-based efforts are one component of a broader, comprehensive approach to addressing youth substance use. CDC’s Evidence-Based Strategies to Prevent Youth Substance Use (ENGAGE) program (https://www.cdc.gov/overdose-prevention/php/interventions/youth-substance-use-prevention.html) includes evidence-based strategies that local, community, state, or other organizations can use to prevent youth substance use. These strategies can be implemented in schools, early childhood home visitation programs, after-school programs, youth substance use treatment services, and primary care visits.

## Limitations

General limitations for YRBS are available in the overview report of this supplement (*6*). The findings in this report are subject to at least five additional limitations. First, substance use behaviors are based on self-report and might be subject to reporting bias and misclassification. Students might not accurately distinguish between substances, particularly across substances with overlapping effects (e.g., stimulants) or appearance. Survey items on prescription opioid misuse refer broadly to prescription pain medications, although examples focused on opioid-containing medications, which might have resulted in overestimates if respondents included nonopioid pain medications. In addition, substance use measures captured any use within the specified reference period and did not include frequency of use, which could be considered for future studies. Second, analytic denominators varied across substance use measures because of missing data for specific items. Missing data resulted from student nonresponse, invalid responses, and survey design procedures used to reduce respondent effort when schools were selected for both national and site-level YRBS samples, in which some students were not asked all national YRBS questions. If responders and nonresponders systematically differed, this could affect the generalizability of estimates. Third, because these analyses were exploratory and included numerous statistical comparisons without adjustment for multiple testing, statistically significant findings, particularly isolated subgroup differences, should be interpreted cautiously. Fourth, some illegal drug products contain adulterants or other drugs unknown to the person using them (e.g., fentanyl), which might have resulted in discrepancies between reported and actual substance use. Finally, urban-rural classification was based on school-level data, and a student’s residence might have differed from the school-level urban-rural classification used in this analysis. Although school-level urban-rural data were available for approximately 95% of students, NCES locale linkage was not conducted for students who attended private schools. Patterns of substance use behaviors might differ systematically between students attending public and private schools; therefore, the findings related to urban-rural differences might not be representative of all U.S. high school students.

## Future Directions

Additional research might be needed to identify structural and contextual factors associated with adolescent substance use and to improve access to local data needed for public health action. Because rural communities are heterogeneous, substance use patterns, prevention resources, and cultural norms might vary significantly across states and local jurisdictions. Future studies could use state and local YRBS data to examine how urban-rural differences vary across regional substance use profiles. Analyses using pooled years of national data could also examine urban-rural differences by demographic characteristics to help identify populations at increased risk for substance use and guide prevention and intervention efforts. To understand the specific contextual factors (e.g., health care access and cultural norms) that might underlie these observed urban-rural differences, linking YRBS data to other area-level indicators might be an important next step. For example, future studies could examine how structural factors, such as retail access to tobacco and alcohol and prevailing community norms, might contribute to the higher prevalences of cigarette, smokeless tobacco, and electronic vapor product use observed among students in rural schools. In addition, assessing differences in the availability, accessibility, and funding of school-based prevention programs and community-level treatment resources could help identify critical gaps in underserved areas. Collectively, this work might enable public health practitioners to design geographically tailored, multicomponent prevention and treatment initiatives to address youth substance use.

## Conclusion

Findings from this report indicate that alcohol, marijuana, and electronic vapor product use remain common among U.S. high school students. Demographic differences in substance use by sex, race and ethnicity, and grade were observed. Students attending rural public high schools had a higher prevalence of several tobacco-related behaviors than students in city and suburb or town public schools. These findings might be used to guide comprehensive and multicomponent programs involving schools, families, and communities to support youth substance use prevention efforts.

## Footnotes

* 45 C.F.R. part 46.114; 21 C.F.R. part 56.114.
